# Adipose tissue-derived stem cells ameliorate hyperglycemia, insulin resistance and liver fibrosis in the type 2 diabetic rats

**DOI:** 10.1186/s13287-017-0743-7

**Published:** 2017-12-19

**Authors:** Naishun Liao, Youshi Zheng, Haihua Xie, Bixing Zhao, Yongyi Zeng, Xiaolong Liu, Jingfeng Liu

**Affiliations:** 1 The United Innovation of Mengchao Hepatobiliary Technology Key Laboratory of Fujian Province, Mengchao Hepatobiliary Hospital of Fujian Medical University, Fuzhou, 350025 People’s Republic of China; 2 Liver Disease Center, The First Affiliated Hospital of Fujian Medical University, Fuzhou, 350007 People’s Republic of China; 3 The Liver Center of Fujian Province, Fujian Medical University, Fuzhou, 350025 People’s Republic of China

**Keywords:** Adipose tissue-derived stem cells, Type 2 diabetes, Liver fibrosis, TGF-β1/SMAD3 signaling pathway

## Abstract

**Background:**

Type 2 diabetes (T2D) is closely associated with liver fibrosis, but no effective treatments are currently available. This study was designed to investigate the therapeutic effects of ADSCs on insulin resistance, hyperglycemia, and liver fibrosis on T2D rats.

**Methods:**

We first established a T2D rat model with liver fibrosis by using the combination of a high-fat diet (HFD), low-dose streptozotocin (STZ), and carbon tetrachloride (CCl_4_). Subsequently, the model rats were administrated by tail vein injection of PBS or ADSCs, respectively. Thereafter, insulin resistance and liver function were assessed by biochemical analysis, ELISA, histopathological examination, and q-PCR assay, respectively. Moreover, the molecular mechanisms of ADSCs on the effect of the TGF-β1/SMAD3 signaling pathway were further analyzed.

**Results:**

Our data showed that ADSC transplantation significantly alleviated insulin resistance and hyperglycemia in the liver-injured T2D rats. We also found that ADSC transplantation could attenuate liver injury by improving liver function and inhibiting pathological changes of liver fibrosis, as well as through downregulation of TGF-β1 and phosphorylated SMAD3 both in vitro and in vivo.

**Conclusions:**

These findings suggested that ADSC transplantation can ameliorate insulin resistance, hyperglycemia, and liver fibrosis via suppressing TGF-β1/SMAD3 signaling, which may provide a potential treatment strategy for liver fibrosis of T2D.

**Electronic supplementary material:**

The online version of this article (doi:10.1186/s13287-017-0743-7) contains supplementary material, which is available to authorized users.

## Background

Type 2 diabetes (T2D) is a growing global health problem, affecting over 336 million people worldwide [1–3]. T2D is a metabolic disorder characterized by chronic hyperglycemia resulting from insulin resistance and impaired insulin secretion [4, 5]. Recent epidemiological and experimental studies have also revealed that T2D is closely associated with long-term damage, dysfunction, and failure of various organs [5, 6]. Among them, chronic liver disease is a major complication of T2D since liver is the main target organ for insulin in regulating myriad metabolic processes such as glycogen storage and gluconeogenesis. Indeed, T2D significantly increases the risk of the entire spectrum of chronic liver diseases, including non-alcoholic liver disease, cirrhosis, and hepatocellular carcinoma [7–9], and the risk of death from chronic liver diseases is increased in patients with T2D [8, 10, 11]. Thus, preventing the progression of T2D may provide a useful strategy for those patients with chronic liver diseases, considering that patients with T2D have a high prevalence of liver diseases.

For the management of T2D, insulin-sensitizing agents including thiazolidinediones and biguanides are required. However, for the management of those type 2 diabetic patients with chronic liver diseases, these drugs are not allowed, because of their impaired liver function and the potential hepatic toxicology of these conventional agents [7]. Hence, the treatment of T2D in patients with chronic liver diseases is complicated, and an effective strategy to prevent both liver injuries and insulin resistance of these patients need urgent investigation.

Regenerative medicine using adipose tissue-derived stem cells (ADSCs) provides a novel strategy for the treatment of various refractory diseases, in view of the fact that ADSCs possess numerous advantages, including their abundant availability, easy obtainability, and their immunomodulatory activity, as well as the ability to self-renew and their multilineage differentiation potential [12, 13]. Previous studies have suggested that ADSC transplantation alleviated the development of chronic liver diseases, including non-alcoholic liver disease [14–16], liver fibrosis [17, 18], and cirrhosis [19–21]. In addition, it has also been suggested that ADSC transplantation ameliorated hyperglycemia in the animal model of T2D [22, 23]. Therefore, whether ADSCs have therapeutic effects on hyperglycemia, insulin resistance, and liver injury in an animal model of T2D with liver fibrosis deserve further investigation.

Here, we established a type 2 diabetic rat model with liver fibrosis induced by the combination of a high-fat diet (HFD), low-dose streptozotocin (STZ), and carbon tetrachloride (CCl_4_), to investigate the therapeutic effects and the molecular mechanism of ADSC transplantation on hyperglycemia, insulin resistance, and liver fibrosis. Our results suggested that ADSC transplantation not only has the ability to reduce hyperglycemia and insulin resistance, but also could be used to ameliorate liver fibrosis in type 2 diabetic rats. Mechanistically, the results also showed that the transforming growth factor beta 1/mothers against decapentaplegic homolog 3 (TGF-β1/SMAD3) signaling pathway was downregulated after ADSC transplantation in the fibrotic liver tissues, and the ADSCs could block the TGF-β1/SMAD3 signaling pathway in hepatic stellate cells (HSCs). Taken together, these findings indicated that ADSC transplantation may offer a new promising approach for type 2 diabetic patients with liver fibrosis.

## Methods

### Animals

Twenty-four adult male Sprague-Dawley rats (weighing 180–200 g) were obtained from the Center for Animal Experiments of Fujian Medical University (license number SCXK min 2012-0002). They were housed at 20–25 °C under a standard 12/12 light-dark cycle with 60% relative humidity. The rats had ad libitum access to food and autoclaved water. All animal procedures were approved by the Animal Ethics Committee of Fuzhou General Hospital (Fuzhou, China).

### Cell isolation and culture

The isolation of ADSCs was performed following our previous reports [12, 14, 15]. Briefly, the subcutaneous adipose tissues in groin were collected from male Sprague-Dawley rats (n = 6), cut into small pieces, and digested with 0.1% type I collagenase (Sigma-Aldrich, St Louis, MO, USA) in α-MEM (Hyclone, Logan, UT, USA) at 37 °C for 60 min. After, collagenase activity was neutralized by α-MEM containing 20% FBS (Gibco, Mulgrave, VIC, Australia), and filtered through a 100-μm cell strainer, and subsequently the cells were resuspended with osmotic lysates (Biyuntian Biological Co., Ltd., Shanghai, China) and incubated at room temperature for 10 min to eliminate the red blood cells. The remaining cells were seeded into T-75 flasks at a density of 1 × 10^6^/mL, and cultured in α-MEM containing 10% FBS, 100 U/mL penicillin (Life Technologies, Grand Island, NY, USA) and 100 μg/mL streptomycin (Life Technologies, Grand Island, NY USA). Cells from the passage 3 were used in the present study. Human hepatic stellate cell line (LX2) was purchased from Bogu Biotech Co., Ltd., (Shanghai, China) and cultured in the complete medium containing RPMI 1640 (Gibco, Mulgrave, VIC, Australia) and 10% FBS (Gibco, Mulgrave, VIC, Australia).

### Co-culture of ADSCs and HSCs

A co-culture system was constructed using a Transwell chamber (Merck Millipore, Billerica, MA, USA), which could be inserted into the wells of six-well plates. LX2 cells were seeded on six-well culture plates at a density of 5 × 10^5^ cells/well, whereas ADSCs were seeded on the membrane (polyethylene terephthalate, pore size, 0.4 μm) at a density of 5 × 10^5^ cells/well in the Transwell chamber. Cells were treated with or without TGF-β receptor 1 inhibitor (LY2157299, 56 nM; Selleck Chemicals Houston, TX, USA), and incubated at 37 °C in a humidified atmosphere containing 5% CO_2_ for 24 h. After that, LX2 cells were collected for further q-PCR and Western blot analysis.

### Animal model and ADSC transplantation

**Fig. 1:**
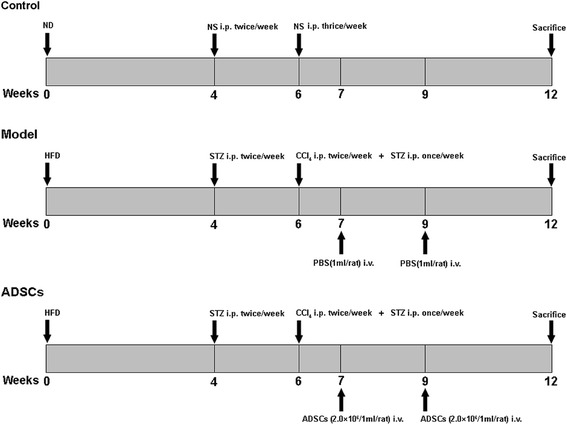
Schematic diagram of the experimental protocol. *ADSCs* adipose tissue-derived stem cells, *HFD* high-fat diet, *ND* normal diet, *NS* normal saline,,*i.p.* intraperitoneal injection, *i.v*. tail vein injection, *STZ* streptozotocin

The T2D model was induced by high-fat diet (HFD) and streptozotocin (STZ) injections as previous descripted with minor modifications [24]. As shown schematically in Fig. 1, 18 Sprague-Dawley rats were fed either normal chow (control group) or a high-fat diet (HFD) containing 66.5% normal chow, 10% lard, 20% sucrose, 2% cholesterol and 1.5% cholate for 4 weeks, followed by intraperitoneal injection of 25 mg/kg dose of STZ (dissolved in the 0.05 M citrate buffer at pH 4.5, prepared immediately before use) twice per week for 2 weeks. After the development of T2D, which was diagnosed as a non-fasting blood glucose ≥11.1 mmol/L by measuring glucose levels in the blood obtained from the tail vein. Diabetic rats with hyperglycemia (n = 12) were randomly divided into two groups: the model group (n = 6), rats were intraperitoneally injected with 20% carbon tetrachloride (CCl_4_) solution in olive oil (3 mL/kg body weight; twice per week for 6 weeks) and 15 mg/kg dose of STZ (once per week for 6 weeks), and treated with tail vein injection of PBS (1 mL/rat) on the weeks 7 and 9; and the ADSC therapy group (n = 6) received intraperitoneal injection of 20% CCl_4_ solution in olive oil (3 mL/kg body weight; twice per week) and 25 mg/kg dose of STZ (once per week), and treated with tail vein injection of ADSCs (2.0 × 10^6^/L mL/rat) on the weeks 7 and 9. After the treatment, all rats were sacrificed with 2% pentobarbital sodium (100 mg/kg; Sigma-Aldrich). The liver tissues (approximately 500 mg/rat) and sera (approximately 3 mL/rat) were collected for further investigation.

### Measurement of blood glucose, insulin and HOMA-IR

After a 12-hour overnight fast, blood from the tail vein was collected and measured by Accuchek Active Meter (ACCU-CHEK® Active; Roche, Berlin, Germany). The insulin levels were analyzed using a rat insulin ELISA kit (BlueGene Biotech Co., Ltd., Shanghai, China) according to the manufacturer’s instruction. Insulin resistance was assessed by the homeostasis model assessment-insulin resistance (HOMA-IR) using the following formula: HOMA-IR = fasting blood glucose (mmol/L) × insulin (mIU/L)/22.5 [25].

### Biochemical assays of liver function

To determine whether ADSC transplantation improved liver function of diabetic rats, the sera were separated after centrifugation at 1000 × g for 10 min at 4 °C, and stored at -80 °C. The serum levels of alanine aminotransferase (ALT) and aspartate aminotransferase (AST), as well as total bilirubin (TBIL) and albumin (ALB) were respectively measured using ALT, AST, TBIL, and ALB assay kits (Nanjing Jiancheng Bioengineering Institute, Nanjing, China), according to the manufacture’s protocol. The serum liver fibrosis indices, including collagen type IV and hyaluronic acid, were measured using rat collagen type IV and hyaluronic acid ELISA kits respectively, according to the manufacturer’s instructions (BlueGene Biotech Co., Ltd., Shanghai, China). The serum levels of pro-inflammatory cytokines including tumor necrosis factor alpha (TNF-α), interleukin 6 (IL-6), and C-reactive protein (CRP) were evaluated using commercial ELISA kits (Boster Biological Technology Co., Ltd., Wuhan, China).

### Hydroxyproline, triglyceride, and total cholesterol content measurement

To further investigate whether ADSC transplantation could improve liver function, the hydroxyproline, triglyceride, and total cholesterol content in liver tissues were measured using commercial kits (Nanjing Jiancheng Bioengineering Institute, Nanjing, China), in accordance with the protocol of the manufacturer’s manual.

### Histopathological assessment

The obtained liver tissues were fixed in 4% paraformaldehyde for 24 h, then gradually dehydrated with ethanol and embedded in paraffin, and finally tissue sections of 5 μm thickness were stained with hematoxylin and eosin (H&E) for histological analysis. To observe the hepatic fibrosis, Masson’s trichrome staining was performed using a commercial kit (Nanjing Jiancheng Bioengineering Institute, Nanjing, China) following the manufacturer’s protocol. To further clarify the fatty infiltration in the liver, frozen liver tissues were sectioned at 10 μm and stained with an Oil Red O staining kit (Nanjing Jiancheng Bioengineering Institute, Nanjing, China) following the manufacturer’s protocol. Double-blind evaluation of hepatic histopathological changes was performed by two expert pathologists. The histopathological examination was performed using an inverted phase-contrast microscope (Carl Zeiss, Oberkochen, Germany), and the fibrosis surface area was analyzed using the ZEN 2012 Light Edition imaging analysis system (Carl Zeiss).

### Quantitative real-time PCR analysis

The total RNA was isolated from liver tissues or LX2 cells using TRIzol reagent (TransGen Biotech Co., Ltd., Beijing, China). Afterwards, the mRNA was reversely transcribed to cDNA by using a transcriptor first-strand cDNA synthesis kit (Roche Applied Science, Mannheim, Germany) according to the manufacturer’s instructions, and q-PCR analysis was performed using the ABI StepOnePlus real-time PCR system (Applied Biosystems Inc., Foster City, CA, USA) with q-PCR Master Mix (DBI Bioscience, Ludwigshafen, Germany). Cycling conditions were as follows: 40 cycles of 95 °C for 15 sec, 60 °C for 30 sec, and 70 °C for 30 sec. Primer sequences are listed in Additional file 1: Table S1. The expression of target gene was normalized to that of β-actin gene. Relative gene expression was calculated with the 2^-△△Ct^ formula.

### Western blot analysis

Samples were lysed in ice-cold RIPA buffer (0.5 M Tris-HCl, pH 7.4, 1.5 M NaCl, 2.5% deoxycholic acid, 10% NP-40, 10 mM EDTA) with protease inhibitor cocktail (Roche, Indianapolis, IN, USA). Protein quantification was performed by BCA assay, and equal amounts of protein lysate (40 μg) were separated by 10% SDS-PAGE. Transfer to nitrocellulose membranes was performed in transfer buffer (12 mM Tris base, 96 mM glycine, pH 8.3, and 15% methanol). Afterwards, the membranes were blocked for 2 hours in the TBST buffer with 5% BSA and probed with the TGF-β1, p-SMAD3, SMAD3 (all from Cell Signaling Technologies, Danvers, MA, USA; 1:500 dilution) and β-actin antibody (Transgen, Beijing, China; 1:5000 dilution) overnight at 4 °C. The membranes were washed with TBST buffer for three times, followed by incubating with appropriate HRP-conjugated secondary antibody (1:5000 dilution; TransGen Biotech Co., Ltd., Beijing, China) for 1 hour at room temperature. Finally, the protein expression levels were detected by enhanced chemiluminescence and visualized by autoradiography.

### Statistical analysis

All quantitative data were expressed as the mean ± standard deviation (SD). All the statistical analyses were performed with GraphPad Prism version 6.0. (GraphPad Software, San Diego, CA, USA), Statistical analysis among different groups was performed using Student *t* test. The *p* < 0.05 was considered as statistically significant.

## Results

### ADSC transplantation ameliorates hyperglycemia and insulin resistance

**Fig. 2:**
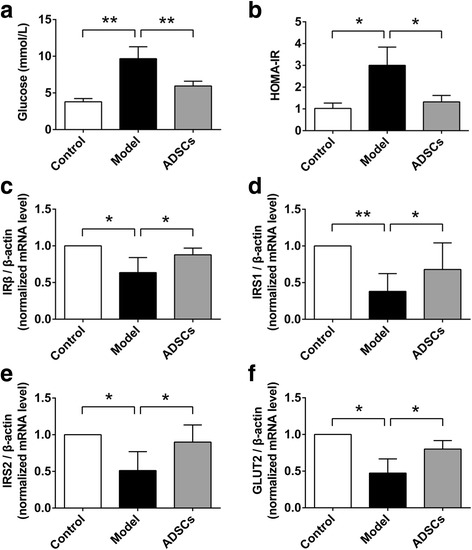
ADSC transplantation inhibits insulin resistance in CCl_4_-injured T2D rats. **a** Serum level of fasting blood glucose after ADSC transplantation. **b** HOMA-IR of T2D rats after ADSC transplantation. The relative mRNA expression of IRβ (**c**) and IRS1 (**d**), as well as IRS2 (**e**) and GLUT2 (**f**) in the liver tissues after ADSC transplantation (*n* = 6 per group; *p < 0.05; **p < 0.01). *ADSCs* adipose tissue-derived stem cells, *GLUT2* glucose transporter 2, *HOMA-IR* homeostasis model assessment-insulin resistance, *IR-β* insulin receptor beta, *IRS1* insulin receptor substrate 1, *IRS2* insulin receptor substrate 2

In order to evaluate the effects of ADSC transplantation on hyperglycemia and insulin resistance, the fasting blood glucose and insulin levels were measured. Compared with the normal (control) rats, markedly higher levels of blood glucose and HOMA-IR were observed in the model group, which means the animal model of T2D was successfully established by using the HFD and STZ. However, after the ADSC transplantation, the levels of blood glucose and HOMA-IR in the ADSCs treatment group were significantly decreased compared to the rats treated with PBS (in the model group) (Fig. 2a, b). These results suggested that ADSC transplantation effectively ameliorated the hyperglycemia and insulin resistance in the CCl_4_-injured type 2 diabetic rats.

To investigate the further mechanisms of ADSC transplantation on insulin resistance, we used a q-PCR analysis method to evaluate the expression of insulin resistance-related genes, including IRβ and IRS1, as well as IRS2 and GLUT2 in the liver tissues. As shown in Fig. 2c–e, the mRNA expression of IRβ, IRS1, IRS2, and GLUT2 was significantly decreased in the CCl_4_-injured diabetic rats; while the gene expression levels were effectively increased after ADSC transplantation in the ADSCs treatment group compared with those in the model group, which indicates the alleviators of the insulin resistance in the insulin-affected liver tissues.

### ADSC transplantation reduces pro-inflammatory cytokines

**Fig. 3:**
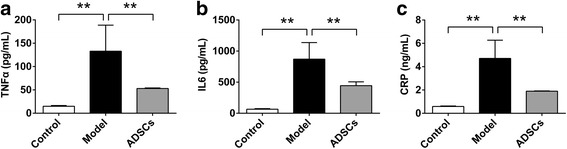
ADSC transplantation reduces serum levels of pro-inflammatory cytokines in CCl_4_-injured T2D rats. Serum levels of TNF-α (**a**), IL-6 (**b**) and CRP (**c**) after ADSC transplantation (*n* = 6 per group; *p < 0.05; **p < 0.01). *ADSCs* adipose tissue-derived stem cells, *CRP* C-reactive protein, *IL-6* interleukin 6, *TNF-α* tumor necrosis factor alpha

Inflammatory processes either contributes to insulin resistance or deteriorates as a consequence of the metabolic dysregulation associated with insulin resistance [26]. To further investigate the protective effects of ADSCs on insulin resistance of T2D rats, the serum levels of pro-inflammatory cytokines including TNF-α, IL-6 and CRP, were also analyzed by ELISA. As shown in Fig. 3, the serum levels of TNF-α, IL-6 and CRP were significantly increased in the model group compared with those in the control group, indicating the model rats were undergoing inflammation; after ADSC transplantation, the decreased serum levels of TNF - α, IL-6 and CRP were clearly observed when compared with those in the model group. These data clearly suggested that ADSC transplantation could reduce inflammation in T2D rats.

### ADSC transplantation improves liver function

**Fig. 4:**
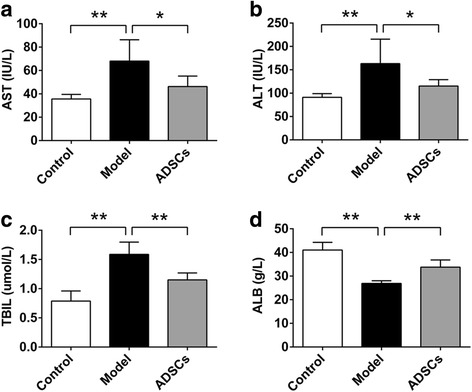
ADSC transplantation improves liver functions in CCl_4_-injured T2D rats. Serum levels of AST (**a**), ALT (**b**), TBIL (**c**), and ALB (**d**) after ADSC transplantation (n = 6 per group; *p < 0.05; **p < 0.01). *ADSCs* adipose tissue-derived stem cells, *ALB* albumin, *AST* aspartate aminotransferase, *ALT* alanine aminotransferase, *TBIL* total bilirubin

The serum levels of AST, ALT, TBIL, and ALB were measured to determine the liver function in the CCl_4_-injured diabetic rats. As shown in Fig. 4, the increased serum levels of AST, ALT and TBIL, and the decreased serum level of ALB were markedly observed in the model group compared with those in the control group, suggesting the serious hepatic damage in the CCl_4_-injured diabetic rats; however, after the ADSC transplantation, the reduced serum levels of AST, ALT, and TBIL, and the increased serum level of ALB could be achieved when compared with those in the model group (PBS-treated rats). Thus, ADSC transplantation effectively improved liver function in the CCl_4_-injured diabetic rats.

### ADSC transplantation reverses histological changes of liver fibrosis

**Fig. 5:**
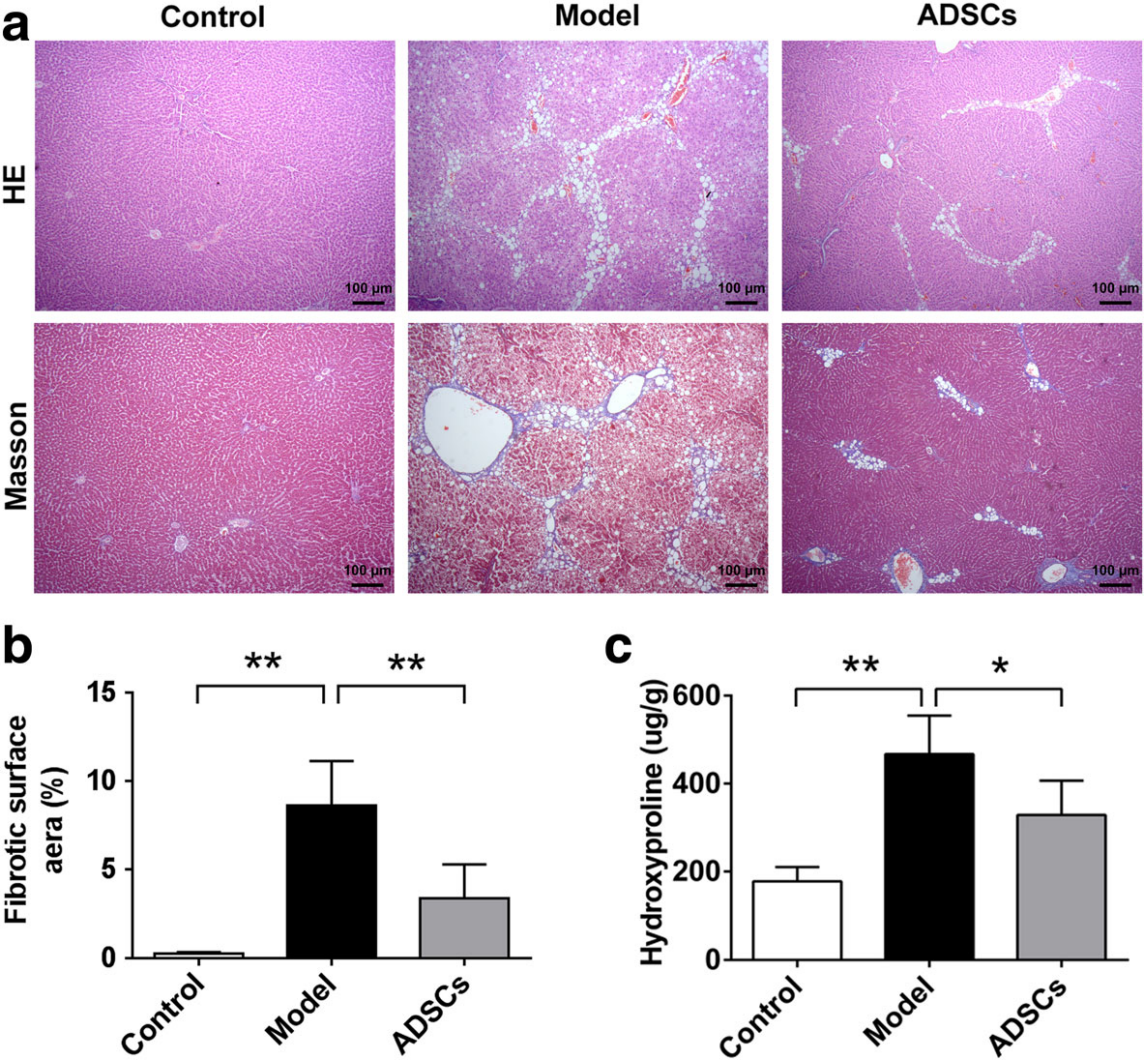
ADSC transplantation reverses pathological changes of hepatic fibrosis in CCl_4_-injured T2D rats. **a** Representative images of liver sections stained with H&E and Masson’s trichrome after ADSC transplantation. The fibrotic surface area (**b**) of Masson’s trichrome stained liver tissues. **c** Hydroxyproline content of liver tissues after ADSC transplantation (*n* = 6 per group; *p < 0.05; **p < 0.01). *ADSCs* adipose tissue-derived stem cells, *H&E* hematoxylin and eosin

The histological examination of the liver tissues was performed by H&E and Masson’s trichrome staining after the injection of ADSCs into the CCl_4_-injured type 2 diabetic rats. As shown in Fig. 5a, the liver tissues from the normal rats exhibited no evidence of inflammation and liver fibrosis, while typical fibrosis and inflammatory infiltration were clearly observed around the central vein of the injured livers in the model rats, which means the animal model of liver fibrosis was successfully established in the type 2 diabetic rats; significantly, there was less evidence of inflammatory infiltration and liver fibrosis in the ADSCs-treated group compared with the model group; moreover, the typical fatty infiltration was observed in the model group, while a slight lipid accumulation was observed in the ADSCs-treated group compared with the model group (Additional file 2: Figure S1a). Taken together, these data suggested that ADSC transplantation could reduce hepatic histological changes in the CCl_4_-injured diabetic rats.

To further determine the degree of liver fibrosis, the data was qualified by analyzing the fibrotic surface area of Masson’s trichrome staining. According to the quantification results, the fibrotic surface area of liver tissue sections in the model group was significantly increased compared with those in the normal rats; but the degree of liver fibrosis could be significantly decreased after ADSC transplantation compared with those in the model group (Fig. 5b), which suggested that ADSC transplantation effectively alleviated the degree of liver fibrosis in type 2 diabetic rats.

Based on the observation of the protective effects of ADSC transplantation on hepatic histology, we next analyzed the hydroxyproline content of liver tissues. Compared with control group, the hepatic hydroxyproline content was significantly increased in the model group; while rats of the ADSCs-treated group exhibited markedly decreased hydroxyproline content compared to those treated with PBS in the model group (Fig. 5c), which suggested that ADSC transplantation could effectively reduce hydroxyproline content in the liver tissues of the CCl_4_-injured diabetic rats.

### ADSC transplantation promotes lipid metabolism

Considering that the lipid accumulation was present in the liver tissues of CCl_4_-injured diabetic rats, we also analyzed the lipid indicators including triglyceride and total cholesterol in the liver tissues. Comparing with the control group, the increased triglyceride and total cholesterol content of the liver tissues were observed in the model group; while the decreased triglyceride and total cholesterol content in the liver tissues were observed in the ADSCs-treated group compared with those in the model group (Additional file 2: Figure S1b, S1c). Thus, our data suggested that ADSC transplantation could also promote lipid metabolism in the liver tissues.

### ADSC transplantation ameliorates liver fibrosis

**Fig. 6:**
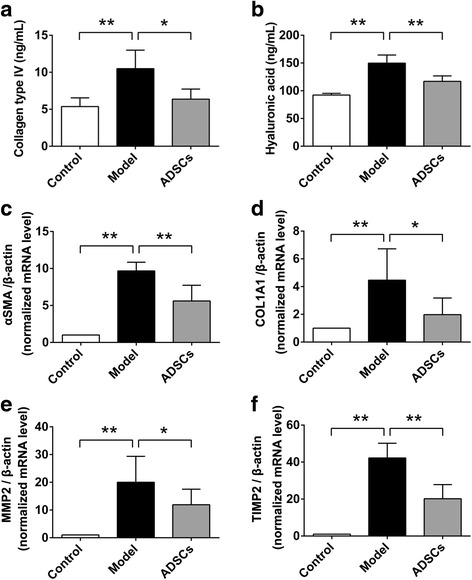
ADSC transplantation alleviates liver fibrosis in CCl_4_-injured T2D rats. Serum levels of collagen type IV (**a**) and hyaluronic acid (**b**) after ADSC transplantation. The relative mRNA expression of α-SMA (**c**) and COL1A1 (**d**), as well as MMP2 (**e**) and TIMP2 (**f**) in the liver tissues after ADSC transplantation (*n* = 6 per group; *p < 0.05; **p < 0.01). *ADSCs* adipose tissue-derived stem cells, *α-SMA* alpha smooth muscle actin, *COL1A1* collagen type I alpha 1, *MMP2* matrix metalloproteinase 2, *TIMP2* tissue inhibitor of metalloproteinases 2

The serum level of liver fibrosis indicators including collagen type IV and hyaluronic acid were analyzed by ELISA. Compared with control group, the serum level of collagen type IV and hyaluronic acid were significantly increased in the model group; while the decreased serum level of collagen type IV and hyaluronic acid were observed in the ADSCs-treated group compared to those treated with PBS in the model group (Fig. 6a, b), which suggested that ADSC transplantation could effectively reduce the serum levels of collagen type IV and hyaluronic acid in the CCl_4_-injured diabetic rats.

In the light of the benefited effects of ADSC transplantation on the histology and serum level of liver fibrosis, we further determined the expression of the hepatic fibrosis-related genes, including alpha smooth muscle actin (α-SMA), collagen type I alpha 1 (COL1A1), matrix metalloproteinase 2 (MMP2), and tissue inhibitor of metalloproteinases 2 (TIMP2), in the liver tissues. As shown in Fig. 6c–f, the mRNA expression αSMA, COL1A1, MMP2, and TIMP2 were significantly increased in the liver tissues of model group compared with those in the control group; while these gene expressions were effectively suppressed after ADSC transplantation when compared with the model group. These results suggested that ADSC transplantation effectively ameliorated the liver fibrosis in the CCl_4_-injured diabetic rats.

### ADSC transplantation suppresses liver fibrosis by inhibiting TGF-β1/SMAD3 signaling

**Fig. 7:**
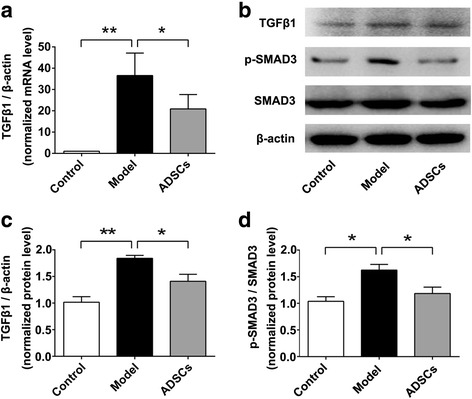
ADSC transplantation ameliorates liver fibrosis by inhibiting TGF-β1/SMAD3 signaling. **a** The relative mRNA expression of TGF-β1 in the liver tissues after ADSC transplantation. **b** Western blot analysis for TGF-β1, p-SMAD3, SMAD3, and β-actin in the liver tissues after ADSC transplantation. Relative expression of TGF-β1 (**c**) and p-SMAD3 (**d**) in the liver tissues after ADSC transplantation (*n* = 6 per group; *p < 0.05; **p < 0.01). *ADSCs* adipose tissue-derived stem cells, *p-SMAD3* phosphorylation of mothers against decapentaplegic homolog 3, *SMAD3* mothers against decapentaplegic homolog 3, *TGF-β1* transforming growth factor beta 1

In order to further explore the underlying mechanisms by which ADSC transplantation suppressed liver fibrosis, we next investigate whether TGF-β1/SMAD3 signaling was involved in the therapeutic effects of ADSC transplantation on liver fibrosis since the TGF-β1/SMAD3 signaling plays an important role in the progression of liver fibrosis [27, 28]. To this end, the mRNA level of TGF-β1 expression in the liver tissue form each groups were determined by q-PCR analysis, and the protein level of TGF-β1, SMAD3, and p-SMAD3 were evaluated by Western blot analysis. As shown in Fig. 7, the relative mRNA and protein levels of TGF-β1, as well as the normalized phosphorylation of SMAD3 (p-SMAD3) were significantly increased in the liver tissues of model group compared with those of the control group, indicating the TGF-β1/SMAD3 signaling was activated in the CCl_4_-injured diabetic rats; however, after ADSC transplantation, the mRNA and protein levels of TGF-β1, and the normalized p-SMAD3 were effectively downregulated compared with those of the model group, suggesting that ADSC transplantation could suppress TGF-β1/SMAD3 signaling of fibrotic liver tissues from CCl_4_-injured diabetic rats.

### ADSCs inhibit TGF-β1/SMAD3 signaling in HSCs

**Fig. 8:**
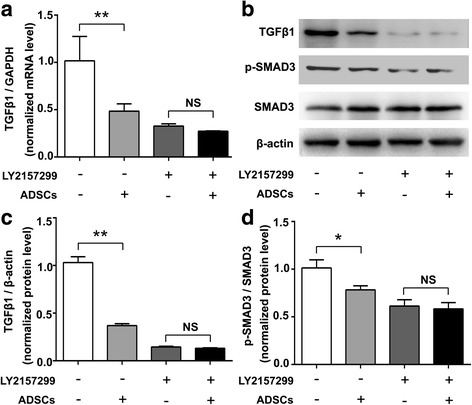
ADSCs suppress TGF-β1/SMAD3 signaling in HSCs. **a** The relative mRNA expression of TGF-β1 in HSCs. **b** Western blot analysis for TGF-β1, p-SMAD3, SMAD3, and β-actin in HSCs. Relative expression of TGF-β1 (**c**) and p-SMAD3 (**d**) in HSCs (*p < 0.05; **p < 0.01). *ADSCs* adipose tissue-derived stem cells, *HSCs* hepatic stellate cells, *p-SMAD3* phosphorylation of mothers against decapentaplegic homolog 3, *SMAD3* mothers against decapentaplegic homolog 3, *TGF-β1* transforming growth factor β1

Considering that TGF-β1/SMAD3 signaling pathway is a key mediator of HSC activation that leads to liver fibrosis [28], we further used a co-culture system of ADSCs together with HSCs to investigate whether ADSCs could suppress TGF-β1/SMAD3 signaling in HSCs. As shown in Fig. 8, the relative mRNA and protein levels of TGF-β1, as well as the phosphorylation of SMAD were significantly inhibited after co-culturing with ADSCs; however, this effect was invalid after further treating with TGF-β receptor 1 inhibitor (LY2157299). These data suggested that ADSCs also blocked TGF-β1/SMAD3 signaling pathway in HSCs.

## Discussion

The liver is the major organ in maintaining glucose homeostasis. It stores glycogen by the uptake of glucose in the postprandial state and produces glucose through glycogenolysis and gluconeogenesis in the postabsorptive state. Hyperglycemia, resulting from the dysregulation of glucose homeostasis, is commonly recognized as the major contributing factor for T2D development. Recently, evidence has also been found that hyperglycemia will in turn promote liver injury through increasing oxidative stress, cytokine levels, and stress signaling pathways, such as the PI3K/Akt and JNK signal pathway [29]. Hence, there are close relationships between chronic liver diseases and imbalance of glucose homeostasis. Additionally, insulin resistance, typically presents from the prediabetes to the later stages of T2D and its complications, plays a major role in the etiology of T2D [30, 31]. In particular, insulin resistance parallels the liver fibrosis stage and is a significant pathophysiological feature of T2D patients with chronic liver diseases [32]. Therefore, hyperglycemia and insulin resistance play a central role in progression of T2D patients with chronic liver diseases. In the current study, the non-fasting blood glucose was significantly increased in our model rats after the typical combination treatment of HFD and STZ for 6 weeks (Additional file 3: Figure S2), indicating the successful establishment of hyperglycemia in T2D. After the following treatment of STZ and carbon tetrachloride for another 6 weeks, hyperglycemia and insulin resistance, as well as injured liver function and liver fibrosis were all presented in our animal model rats, implying that the animal model of liver fibrosis in T2D was successfully established in the current study.

Considering that hyperglycemia and insulin resistance have a significant role in the progression of liver injuries of T2D, effective treatments targeting hyperglycemia and insulin resistance are extremely necessary for the successful treatment of those patients. Although insulin sensitizing agents including thiazolidinediones and biguanides have been clinically used in the majority of T2D, the treatment of the diabetic patients with chronic liver diseases is inapplicable, because of the impaired liver function and the potential hepatotoxicity of those conventional drugs. As a result, to date, it is still no satisfactory therapeutic treatment for the diabetic patients with liver diseases. Regenerative medicine using ADSCs is being considered as alternative therapeutic approach for treating T2D alone, and chronic liver diseases including non-alcoholic liver diseases, liver fibrosis, and cirrhosis [14–22]. However, whether ADSC transplantation has a therapeutic effect on chronic liver diseases of T2D remains to be fully elucidated. In the current study, ADSC transplantation was able to alleviate hyperglycemia and insulin resistance, recover the injured liver function, and protected against the pathogenic changes of liver fibrosis of type 2 diabetic rats, which suggested that ADSC transplantation may be an effective therapeutic approach for T2D patients with liver fibrosis.

It is widely acknowledged that abnormal expression of TGF-β1 is involved in the process of liver fibrosis, and the fibrogenic functions of TGF-β1 are mainly mediated by the canonical SMAD pathway via activation of transmembrane TGF-β1 receptors [33, 34]. Thus, to further explore the therapeutic target of ADSCs for treating liver fibrosis, we investigated the expression of key mediators of TGF-β1/SMAD signaling including TGF-β1, SMAD3 and p-SMAD3. As predicted, the upregulation of TGF-β1 and p-SMAD3 was observed in the CCl_4_-injured type 2 diabetic rats, while the expression of TGF-β1 and p-SMAD3 was blocked after ADSC transplantation, suggesting that ADSC transplantation effectively inhibits TGF-β1/SMAD3 signaling in the fibrotic liver tissues. Additionally, we also found that ADSCs could downregulate the expressions of TGF-β1 and p-SMAD3 in HSCs. Taken together, these findings indicates that downregulation of TGF-β1/SMAD3 signaling is a potential mechanism by which ADSC transplantation ameliorates liver fibrosis of T2D.

Here, we showed that ADSC transplantation could effectively ameliorate hyperglycemia, insulin resistance, and liver fibrosis. However, the clinical application of our approach is somewhat limited since the safety of ADSCs has not yet been clearly elucidated. Further studies are necessary to ascertain this information before the clinical application of our approach. Nevertheless, we clearly demonstrates that ADSC transplantation exert a therapeutic effects on liver fibrosis of T2D, a finding that may provide a new promising approach for treating T2D patients with liver fibrosis.

## Conclusions

In conclusion, we demonstrated that ADSC transplantation effectively inhibits hyperglycemia and insulin resistance, and alleviates liver fibrosis in the T2D rats. Therefore, ADSC transplantation presents a new therapeutic strategy for treating T2D patients with intractable liver fibrosis.

## Additional files

Additional file 1: Table S1. Primers for q-PCR. (DOC 41 kb)

Additional file 2: Figure S1. ADSC transplantation promotes lipid metabolism in the liver tissues of T2D rats. (**a**) Oil Red O staining of liver tissues in CCl_4_-injured T2D rats. The content of triglyceride (**b**) and total cholesterol (**c**) in the liver tissues after ADSC transplantation (*n* = 6 per group; **p < 0.01). *ADSCs* adipose tissue-derived stem cells. (TIF 8166 kb)

Additional file 3: Figure S2. The non-fasting blood glucose after the combination treatment of HFD and STZ for 6 weeks. (*n* = 6 per group; **p < 0.01). *HFD* high-fat diet, *STZ*, streptozotocin. (TIF 135 kb)

## Abbreviations

ADSCs: Adipose tissue-derived stem cells
ALB: Albumin
ALT: Alanine aminotransferase
AST: Aspartate aminotransferase
CCl_4_: Carbon tetrachloride
COL1A1: Collagen type I alpha 1
CRP: C-reactive protein
GLUT2: Glucose transporter 2
HFD: High-fat diet
HOMA-IR: Homeostasis model assessment-insulin resistance
HSCs: Hepatic stellate cells
IL-6: Interleukin 6
IRS: Insulin receptor substrate
IRβ: Insulin receptor beta
MMP2: Matrix metalloproteinase 2
SMAD3: Mothers against decapentaplegic homolog 3
STZ: Streptozotocin
T2D: Type 2 diabetes
TBIL: Total bilirubin
TGF-β1: Transforming growth factor beta 1
TIMP2: Tissue inhibitor of metalloproteinases 2
TNF-α: Tumor necrosis factor alpha
αSMA: Alpha smooth muscle actin
