# Supplementary material for: Adipose tissue-derived stem cells ameliorate hyperglycemia, insulin resistance and liver fibrosis in the type 2 diabetic rats
Source: Stem Cell Res Ther. 2017 Dec 19;8:286. doi: 10.1186/s13287-017-0743-7 (PMC5738093; doi:10.1186/s13287-017-0743-7)
Supplement: Supplementary file 1 — Primers for q-PCR. (DOC 41 kb) [file 13287_2017_743_MOESM1_ESM.doc]

**Table S1. Primers for q-PCR**

| Gene | Species | Forward primer | Reverse primer |
| --- | --- | --- | --- |
| *β-actin* | rat | atagcacagcctggatagcaacgtac | caccttctacaatgagctgcgtgtg |
| *IRβ* | rat | ttcgaggagagaccttggaa | tcgtgaggttgtgcttgttc |
| *IRS1* | rat | tggacaaacggagtaggg | ctggtggaag aggaggaa |
| *IRS2* | rat | caagcagatcctgcagccacg | gttctccatagacagcttggag |
| *GLUT2* | rat | ctgggtctgcaatttcatca | cgtaaggcccgaggaagt |
| *αSMA* | rat | ccatcaggaacctcgagaagc | agctgtccttttggcccatt |
| *COL1A1* | rat | cctcaaggtttccaaggacc | caatccatccagaccgttgtg |
| *MMP2* | rat | tcccgagatctgcaagcaag | agaatgtggccaccagcaag |
| *TIMP2* | rat | gacacgcttagcatcacccaga | ctgtgacccagtccatccagag |
| *TGFβ1* | rat | ggactctccacctgcaagac | gactggcgagccttagtttg |
| *TGFβ1* | human | ggccagatcctgtccaagc | gtgggtttccaccattagcac |
| *GAPDH* | human | agccacatcgctcagacac | gcccaatacgaccaaatcc |
