# Supplementary figures and images for: Adipose tissue-derived stem cells ameliorate hyperglycemia, insulin resistance and liver fibrosis in the type 2 diabetic rats
Source: Stem Cell Res Ther. 2017 Dec 19;8:286. doi: 10.1186/s13287-017-0743-7 (PMC5738093; doi:10.1186/s13287-017-0743-7)

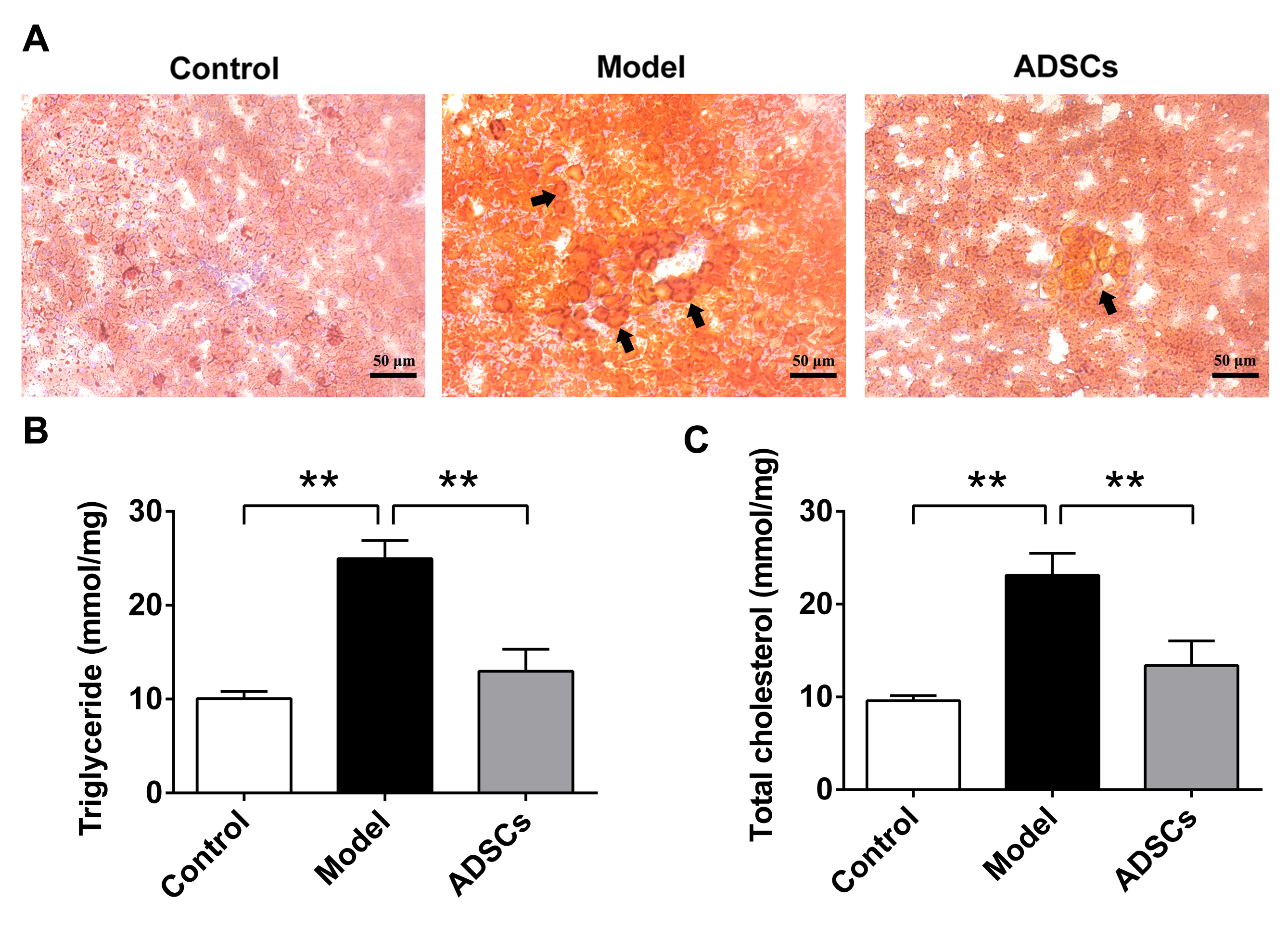

Supplement: Supplementary file 2 — ADSC transplantation promotes lipid metabolism in the liver tissues of T2D rats. (a) Oil Red O staining of liver tissues in CCl4-injured T2D rats. The content of triglyceride (b) and total cholesterol (c) in the liver tissues after ADSC transplantation (n = 6 per group; **p < 0.01). ADSCs adipose tissue-derived stem cells. (TIF 8166 kb) [file 13287_2017_743_MOESM2_ESM.tif]
